# *Artemisia* Allergy Research in China

**DOI:** 10.1155/2015/179426

**Published:** 2015-04-27

**Authors:** Rui Tang, Jin-Lu Sun, Jia Yin, Zhi Li

**Affiliations:** Department of Allergy, Peking Union Medical College Hospital, Peking Union Medical College and Chinese Academy of Medical Sciences, Beijing 100730, China

## Abstract

*Artemisia* is the most important outdoor allergen throughout China. It can cause allergic rhinitis, asthma, or both of them. Since it was verified as an allergenic pollen in 1960, it was identified two times in the Chinese National Pollen Survey (1984, 2009). The first oral immunotherapy double-blinded trial for *Artemisia* pollen asthma research was conducted in China in 1989 and published in 1990. 40 years since that study, there have been many published research reports on Chinese *Artemisia* allergy. This review summarizes the information regarding the discovery of *Artemisia* as an allergenic pollen, pollen account, epidemiology, allergen components, immunological changes in hay fever patients, natural course from rhinitis to asthma, diagnosis, and immunotherapies in China.

## 1. Introduction

*Artemisia* species, or mugwort, is an anemophilous genus included in the Compositae family. Mugwort plants produce high pollen grain quantities [1–4]. This plant is characterized by a huge production of small pollen grains that can be transported for several hundreds or even thousands of kilometers by large air masses. The occurrence of* Artemisia* species is associated with dry or slightly moist habitats and full exposure to light, which are important components of steppes [5].

Pollen from the various* Artemisia* species is one of the most frequent and serious pollinosis causes in many parts of the world [5–9]. The genus* Artemisia* includes 57 species in Europe [10] and 187 species in China [11]. In this review, we summarize* Artemisia* allergy research in China.

## 2. Discovery of* Artemisia* as an Allergenic Pollen

In the 1950's, Professor Ye in ENT Department of Peking Union Medical College Hospital (PUMCH) had finished his allergy practice training in Johns Hopkins Hospital in USA and learnt that ragweed was the main allergenic pollen in autumn in Europe and United States. He found that many allergy patients showed negative reaction with ragweed prick skin test. Confusingly, there were too many allergic rhinitis patients visiting his clinic every autumn. After then, Ye [12] found that there was a type of weed,* Artemisia annua*, that grew widely in North China. Further, they found that the most abundant pollen count dates were September 4, August 24, and September 5 in 1962, 1963, and 1972, respectively. The allergic patient numbers and their symptom severities were related to the local pollen counts. By a nasal challenge test with* Artemisia annua* extract, this study verified that* Artemisia annua* is a major outdoor allergen source in North China.

To clarify the allergenicity of the nonpollen containing components of the plant, they collected and extracted* Artemisia annua* leaves and stems before the pollination period in 1987 [13]. They showed that the pollen-free plant extracts did have in vivo allergenic activities.

## 3. *Artemisia* Allergic Rhinitis and Airway Hyperresponsiveness

To study the relationship between* Artemisia* allergic rhinitis and airway hyperresponsiveness, Ma et al. [14] chose 50* Artemisia* hay fever patients and 20 normal controls. Each of these subjects was separately engaged with a skin test with* Artemisia annua* extract. All 50 patients had an intensely positive reaction to the* Artemisia annua* pollen skin test, but all of the controls were negative. Additionally, they conducted a bronchial provocation test (BPT) with a 1 : 100 (w/v)* Artemisia annua* pollen dilution. Before and after the BPT, they separately tested the forced expiratory volume in first second (FEV1) and serum eosinophil cationic protein (ECP) levels. They concluded that patients with* Artemisia* allergic rhinitis not only had chronic inflammation in their nose mucosa that resulted from an allergic reaction but also had chronic bronchial inflammation, leading to airway hyperresponsiveness (AHR). Their results also reveal that most patients with allergic rhinitis have AHR (76%).

## 4. Features of Pollen Prevalence

### 4.1. National Pollen Surveys

**Figure 1:**
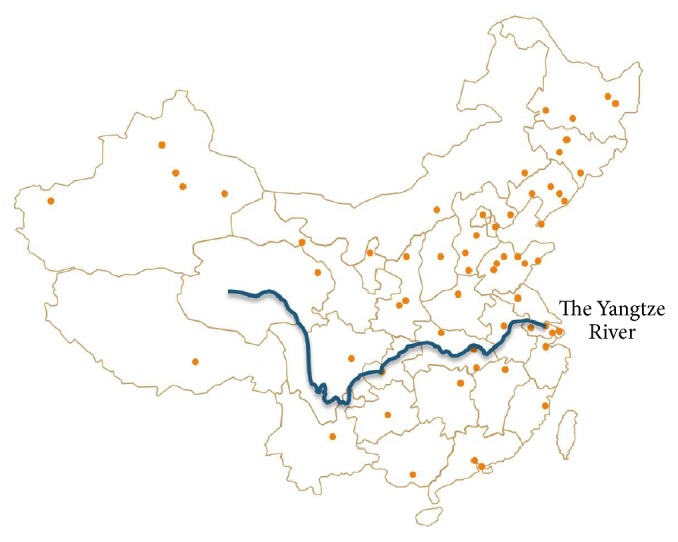
Distribution of* Artemisia* pollen in China.

From 1984 to 1989, Ye [15], at PUMCH, led the first national pollen survey in mainland China. They found that* Artemisia* pollen could be counted in each province in mainland China (Figure 1). At the same time, they got* Artemisia* pollen count data from many Chinese cities in 1988 autumn (Table 1). In 2009, Yin, at the same allergy department, led the second national epidemic allergy study from 100,000 populations in 18 provinces and cities in mainland China, and that data will be published soon.

### 4.2. Other Pollen Count Studies

During the periods that occurred between 1983 and 1986 and between 1999 and 2007, daily airborne pollen monitoring was performed by the gravitational method, using Ye's sampler at the top of the Peking Union Medical Hospital Outpatient Department Building, Beijing, China. He et al. [16] found that* Artemisia* was the most abundant airborne pollen and showed that it was produced over the longest period within a year, from the beginning of July until the end of September. The* Artemisia* levels were followed by* Humulus* pollen levels. Similar reports were conducted with the Durham gravity method in Xi An city (northwestern region of China) [17] in the 1980's and in Wuhan city (central region of China) in the 1990's [18]. From March 31, 2001–April 1, 2002, pollen counts were evaluated in Nanchang city (central region of China). Additionally, Xie et al. [19] found that the highest airborne presence (the percent of total yearly pollen counts) was from* Ambrosia* (35.73%), followed by* Pinaceae* and* Artemisia* (11.94%).

The Burkard volumetric trap was used to sample airborne pollen in Beijing city from August 1, 2007 to October 10, 2007. Yao [20] determined that (1)* Artemisia* and* Humulus* (including* Cannabis sativa* L.) were the main airborne pollen types observed during August and September in Beijing city, which accounted for 31% and 51% of the total pollen levels, respectively; (2) the* Artemisia* pollen season was from August 8th to October 8th; (3) the daily peak mugwort pollen concentration was 267 grain/m^3^, with an average of 71 g/m^3^; and (4) 88.5% of the outpatients that suffered from hay fever or asthma during the Autumn season were allergic to* Artemisia*. This was the first time that the Burkard volumetric sampler was employed for* Artemisia* and* Humulus* concentration monitoring in Beijing city.

In 2005, Qiao et al. [4] published a color atlas of airborne pollens and plants that are prevalent in China. In this book, the main* Artemisia* species on the China mainland were described, which included* Artemisia argyi *Levl. Et Vant.,* Artemisia sieversiana *Willd.,* Artemisia annua *L.,* Artemisia capillaris *Thunb., and* Artemisia lavandulaefolia *DC.

## 5. Epidemiology of* Artemisia* Allergy

A cross-sectional survey was performed that included 6,304 patients who suffered from asthma and/or rhinitis in 17 cities across China [21]. These patients completed a standardized questionnaire that determined their respiratory and allergy symptoms. They also underwent skin prick tests with 13 common aeroallergens. The overall prevalence of the positive skin prick responses was 11.3% for* Artemisia vulgaris*, 6.5% for* Ambrosia artemisiifolia*, 3.5% for mixed grass pollen, and 2.2% for mixed tree pollen. The severity of rhinitis and asthma was significantly correlated with the skin reactivity index to* Artemisia vulgaris* and* Ambrosia artemisiifolia* and to* D. pteronyssinus*,* D. farinae*, and* Blomia tropicalis* (*P* < 0.001). The main pollen and spore families in Beijing are* Artemisia genus*,* Ambrosia genus*, Chenopodiaceae, and Gramineae. They can reach approximately 307,000 grains of pollen/1000 m^3^ of air in August [6].

A total of 215,210 tests with the ImmunoCAP system were assayed in the past three years in the PUMCH Allergy Department, which is the largest allergy department in China, and 76% were inhalant allergens. Among these allergens, the three most prevalent allergens were* Dermatophagoides pteronyssinus, Dermatophagoides farinae*, and* Artemisia* [22].

**Figure 2:**
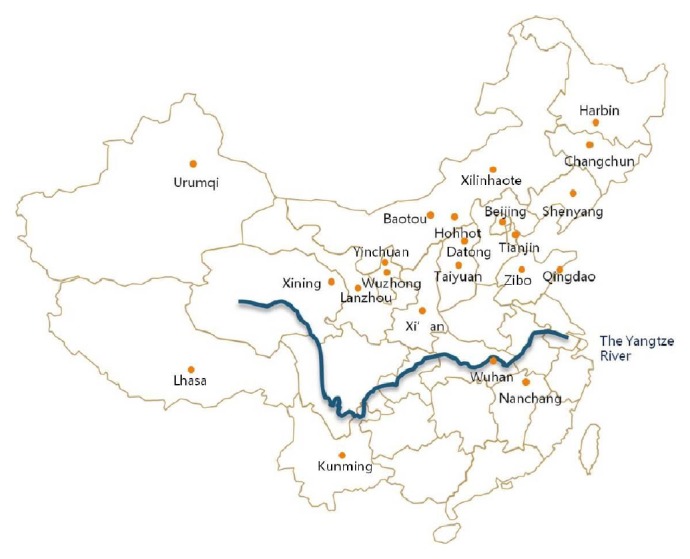
*Artemisia* pollen was the most allergenic pollen in north part area of Yangtze River in China (with skin test or sIgE blood test).

Many studies had been done about* Artemisia* pollen survey and its relationship with allergic diseases in China. The authors showed that* Artemisia* pollen was the most allergenic pollen in northern part area of Yangtze River in China (Figure 2) [23–64].

A total of 1,144 subjects (aged from 5 to 68) from June to October 2011 underwent intradermal testing using a panel of 25 allergen sources [65]. Of the 1,144 subjects, 170 had positive intradermal reactions to pollen and 144 donated serum for IgE testing from these 170 subjects. The positive intradermal response prevalence to* Artemisia sieversiana, Artemisia annua, Ambrosia artemisiifolia*, and* Humulus scandens* pollen was 11.0%, 10.2%, 3.7%, and 6.6%, respectively. Among the intradermal positive subjects, the specific IgE antigen prevalence to* Artemisia vulgaris* was 58.3%, to* Ambrosia artemisiifolia* was 14.7%, and to* Humulus scandens* was 41.0%. The specific IgE antigen prevalence to the Art v 1 allergen was 46.9% and to the Amb a 1 allergen was 11.2%. The correlation between the presence of IgE antibodies that were specific to* Artemisia vulgaris* and to the Art v 1 antigen was very high. Subjects with* Ambrosia artemisiifolia* specific IgE also had* Artemisia vulgaris* specific IgE but with relatively high levels of* Artemisia vulgaris* IgE antibodies. There were no correlations between the presence of IgE antibodies that were specific to* Humulus scandens* and* Artemisia vulgaris*. They concluded that the specific IgE antibody correlations suggest that pollen allergens from* Artemisia* and* Humulus* are independent sources for primary sensitization.

## 6. *Artemisia* Allergens

In 1992, Ou [66] purified the major allergen, A2c, from a wild* Artemisia* sieversiana extract. Its molecular weight was 31 kDa, and its pI was 5.3 kDa and 6.35 kDa. We verified this finding by evaluating the components of wild* Artemisia sieversiana* allergen extract with two-dimensional electrophoresis analyses [67].

To isolate and identify the* Artemisia argyi* and* Artemisia apiacea* pollen, Yang et al. [68] precipitated* Artemisia argyi* and* Artemisia apiacea* pollen extract with saturated ammonium sulfate and then evaluated it with SDS-polyacrylamide gel electrophoresis (SDS-PAGE). They identified the major and minor allergens by western blot analysis. Specifically, they found more than twenty protein bands and 9 allergens in the* Artemisia argyi* pollen extract. The major allergens were 62 kDa, 43 kDa, and 38 kDa. Similarly, in the* Artemisia apiacea* pollen extract, there were 11 allergens. The major allergens were 43 kDa and 38 kDa. The pollen from both species shares many allergens. Unique allergen protein bands in each of the pollen allergens were also identified.

Wu [69] analyzed* Artemisia* and ragweed extract antigens with SDS-PAGE and western blot analysis. They found that there were 13 bands between 18 kDa and 100 kDa in the* Artemisia* extract and 5 bands between 18 kDa and 63 kDa in the ragweed extract. Additionally, cross-reactivity between the* Artemisia* and ragweed extracts existed in the 18 kDa protein.

## 7. Immunological Characteristic in* Artemisia* Hay Fever Patients

### 7.1. The Th1 and Th2 Balance

Qiu et al. [70] collected tonsil lymphocytes in* Artemisia* pollen allergic patients and nonatopic people with a lymphocyte separation medium and incubated these cells with* Artemisia* pollen antigen. They found that the levels of IL-4 and IL-5 in the tonsil lymphocytes of the* Artemisia* pollen allergic patients were higher than those in the nonatopic controls after they were stimulated with this specific antigen, but the levels of IFN-*γ* were lower than those in nonatopic controls (*P* < 0.01). Obvious proliferation for the levels of IL-4 and IL-5 was observed in the allergic group after lymphocytes were stimulated with the specific antigen (*P* < 0.01), but there were no significant changes in the control group. The authors demonstrated that cytokines from Th cells of tonsil lymphocytes from* Artemisia* pollen-allergic people after they were specifically stimulated by* Artemisia* pollen antigen became imbalanced, indicating that Th1 drifts to Th2.

### 7.2. Basophils

With 119 hay fever patients who were allergic to* Artemisia* and 30 nonallergic patients, Zhang et al. [71] found that basophil numbers (mucosal mast cells) in the nasal mucosa as well as nasal eosinophil numbers increased during the pollen season and decreased during the nonpollen season. They concluded that basophils in the nasal mucosa and nasal eosinophils were related to stimulation by* Artemisia* in hay fever patients.

### 7.3. ICAM-1

Eleven patients with* Artemisia* allergic rhinitis were evaluated by Wang et al. [72]. Among them, 8 were studied during pollen season and 3 were studied outside of pollen season. Intercellular adhesion molecule-1 (ICAM-1) was detected on nasal epithelial cells by reverse transcription polymerase chain reaction (RT-PCR). The results showed that ICAM-1 was detectable in all of the pollen season samples. However, during the nonpollen season, 2 of the 3 samples were negative and 1 was positive (this subject was also positive to house dust). It was suggested that ICAM-1 is detectable on nasal epithelial cells during exposure to specific allergens (*Artemisia*).

### 7.4. HLA-DR

To investigate whether susceptibility or resistance to* Artemisia* allergic rhinitis is associated with HLA-DRB alleles or not, Xing [73] tested the frequency distribution of HLA-DRB alleles in 41 patients with* Artemisia* allergic rhinitis (AR) and in 41 healthy controls from Beijing, China, using PCR-SSP (sequence-specific primer polymerase chain reaction). The frequency of HLA-DRB1^∗^ 0301.2 and HLA-DRB4^∗^ 0101 was lower in the AR subjects than the controls (2.44% versus 17.07%, *P* < 0.05; 29.27% versus 51.22%, *P* < 0.05). They showed that the HLA-DRB1^∗^ 0301.2 and HLA-DRB4^∗^ 0101 alleles might confer protection against AR.

To determine whether alleles at one or more of the HLA loci were associated with* Artemisia* pollen hypersensitivity in allergic rhinitis patients, Xing et al. [74] also tested the frequency distribution of the HLA-DQA1 and DQB alleles in 41 patients with allergic rhinitis (AR) and in 41 healthy controls from Beijing with PCR-SSP. They demonstrated that the frequency of HLA-DQA1^∗^ 0201 and DQB1^∗^ 0602 was lower in the AR subjects than the controls (24.39% and 4.88% versus 46.34% and 26.83%, resp.) and the frequency of DQA1^∗^ 0302 was increased among the AR patients (58.54% versus 14.63%). They concluded that the HLA-DQA1^∗^ 0201 and DQB1^∗^ 0602 alleles might confer protection against AR and that DQA1^∗^ 0302 may be an* Artemisia* pollen hypersensitivity susceptibility factor.

## 8. The Natural Course from Rhinitis to Asthma

A total of 1,096 patients with autumnal pollinosis, which excluded those with typical seasonal rhinitis or asthma symptoms but with positive skin tests and serum IgE specific to dust mites and fungi, included 511 with pure allergic rhinitis and 585 with allergic rhinitis complicated with asthma. These subjects underwent inhalant allergen skin tests, evaluations for serum IgE specific to autumnal pollens, and a questionnaire survey. Yin et al. [75, 76] found that the average onset age of the allergic rhinitis patients induced by autumnal pollens was 27.9 years, and this age was significantly younger than that of the allergic asthma patients (32.6 years, *P* < 0.001). Out of the 1,120 patients, 1,096 (97.9%) had allergic rhinitis, 602 (53.8%) had asthma, 507 (45.3%) only had allergic rhinitis, and 10 (0.9%) only had allergic asthma. Among the 1,096 allergic rhinitis patients, 585 (53.4%) suffered from seasonal asthma. Among the 602 asthma patients, 585 (97.2%) suffered from seasonal rhinitis and 183 of the 602 patients (30.8%) needed emergency treatment. The authors showed that autumnal pollens are very important inducers of asthma during the autumn season in northern China and that almost half of the patients with autumnal pollen allergic rhinitis develop seasonal allergic asthma within 9 years [75–77].

From July 1 to October 31, 2006, Wen also observed 18 patients with only allergic rhinitis and 31 patients with allergic rhinitis and asthma [77]. The authors reported that there was a significant correlation between the* Artemisia* pollen count and the scores for night and daytime asthma symptoms, PEF, and diurnal variation in PEF (rs = 0.762, rs = 0.682, rs = −0.649, rs = −0.596, rs = 0.549, *P* < 0.001). It was also concluded that* Artemisia* pollen could trigger autumnal asthma in northern China.

## 9. Diagnostic

To evaluate the value of intradermal skin test (IDT) and serum sIgE detection in diagnosing* Artemisia* sensitivity, 1,150 patients with autumnal rhinitis or asthma were evaluated by experienced physicians. These subjects then underwent IDT with a 1 : 1,000 dilution of (W/V)* Artemisia annua* extract [78]. Then, all patients were examined for* Artemisia* sIgE (w6). The diagnostic standards were established based on the IDT and sIgE results. A reference standard was established according to the typical history and symptoms and a wheal with a diameter ≥ 5 mm and a sIgE level ≥ 0.35 kU(A)/L; a wheal with a diameter ≥ 10 mm alone; or a sIgE level ≥ 0.70 kUa/L alone. When using the reference standard as the criteria, the IDT had better sensitivity (96.2%), specificity (74.2%), positive predictive value (+PV, 93.5%), negative predictive value (−PV, 85.7%), and efficiency (91.6%) than using the sIgE ≥ 0.35 kUa/L alone as the IDT criteria. Additionally, the sIgE detection had better sensitivity (97.6%), specificity (94.9%), +PV (98.7%), −PV (91.1%), and efficiency (97.0%) than using wheal diameter ≥ 5 mm alone as the sIgE detection criteria. The IDT false positive and sIgE detection rates decreased from 35% and 22.7% to 25.6% and 5.1%, respectively, when using a wheal diameter ≥ 10 mm or sIgE ≥ 0.70 kUa/L as the positive criteria.

It was showed that IDT and sIgE detection were well correlated with each other in diagnosing* Artemisia* pollinosis, whereby both of them had the possibility of being false positive, but IDT had a higher false positive rate than sIgE detection. The IDT and sIgE detection false-positive rates can be decreased by increasing the positive criteria to a higher grading criterion.

Sera from 50 weed pollen-induced allergic rhinitis patients were tested for specific serum IgE reactivity against allergenic* Artemisia* extracts (*Artemisia vulgaris*, Art v) and single Art v 1 or Art v 3 allergens [79]. Sera from 88% of the patients demonstrated a positive specific IgE reactivity to Art v, and of these, 82% were positive to Art v 1. The authors found that specific IgE reactivity towards the major mugwort allergen, Art v 1, was a good indicator for Art v sensitization.

## 10. Immunotherapy

### 10.1. Intradermal Immunotherapy

A one-year controlled trial for an immunotherapy was conducted in 50* Artemisia* sensitive hay fever patients (treatment group) [80]. From October 1985 to July 1986, all of the treatment group patients received regular* Artemisia* pollen allergen extract injections over one year, which totaled 30,000 protein nitrogen units (PNU). For these patients, the symptom score indices of the posttreatment 1986 pollination season were compared with those from the pretreatment 1985 season and also with the scores of a similar group of 30* Artemisia* sensitive patients treated only with symptomatic medications during the 1986 season (control group). The 1986 symptom scores for the treatment group were significantly improved, and the effective rate was 78%. An immunological study with the human basophil degranulation test (HBDT) showed a significant decrease in degranulation reactions after immunotherapy. Moreover, the decline in the HBDT positive rate in the treatment group was significantly greater in the patients with improved symptoms than the patients with unchanged symptoms. No difference was observed in basophil degranulation in those patients tested with a pollen-free plant extract, which was not applied in the immunotherapy. The results suggested that immunotherapy could induce basophil desensitization and that the induction might be allergen specific. Basophil desensitization may play an important role in immunotherapy mechanisms.

### 10.2. Oral Immunotherapy

In 1989, eighteen asymptomatic* Artemisia* pollen asthma patients with normal pulmonary functions were selected for a double-blinded oral immunotherapy trial [81]. Each patient had a positive* Artemisia* pollen extract skin test and also had a positive bronchial challenge response to the same extract. The patients were randomly assigned to an active treatment or a placebo group and received intensive oral administration of* Artemisia* pollen extract over a 50-day course. The nine patients who received the active treatment ingested a cumulative dose of 396,652 PNU and showed a significant decrease in serum-specific IgE antibodies (*P* ≤ 0.05) and a significant reduction in bronchial sensitivity to the same extract (*P* ≤ 0.01). The changes in these two variables correlated well. The nine patients who received the placebo showed no significant changes in serum-specific IgE or bronchial sensitivity to the* Artemisia* pollen extract. Follow-ups for two cases with the same extract showed that the reductions in serum-specific IgE as well as bronchial sensitivity induced by oral immunotherapy were maintained for 3 months.

Clinically, sublingual immunotherapy (SLIT) that uses allergen extracts effectively alleviates allergic rhinitis and asthma symptoms. Ma et al. [82] hypothesized that the oral administration of a high dose of allergen extracts imitates SLIT and may prevent IgE-related responses in allergic diseases. In this study, they investigated the effects of the oral administration of mugwort (*Artemisia*) pollen (MP) allergen extracts on allergen-induced inflammation and airway hyperresponsiveness (AHR) in an allergic mouse model. After the administration of MP drops containing Art v 1 and Art v 4 extracts derived from MP specifically in MP-sensitized mice, the effects of the MP drops on AHR, inflammatory cell accumulation, cytokine production in the bronchoalveolar lavage fluid and lung tissue, and serum IgE and IgG levels were investigated. The results indicated that the MP drops not only prevented AHR in response to methacholine in a dose-dependent manner but also significantly reduced the total serum and allergen-specific IgE levels. All of the maximal effects were achieved at a dose of 100 *μ*g/(kgd) and were comparable to the effects of dexamethasone at a dose of 0.5 mg/(kgd). Furthermore, the oral administration of the MP drops dose dependently elevated allergen-specific serum IgG2a levels, reduced total and allergen-specific IgE levels, and normalized the imbalance between the Th1 cytokine IL-12 and the Th2 cytokines IL-4 and IL-5. Finally, the oral administration of the MP drops significantly reduced goblet cell hyperplasia and eosinophilia in the MP-sensitized allergic mouse model. These sets of data suggest that the MP drops effectively improve specific allergen-induced inflammation and AHR in MP-sensitized and MP-challenged mice and provide the rationale for the clinical use of MP drops in specific allergen-induced asthma. Currently, a Stage I clinical SLIT trial for* Artemisia annua *L. was permitted by the Chinese Food and Drug Administration for one Chinese pharmaceutical company (http://www.sfda.gov.cn/WS01/CL0001/).

## 11. Summary

*Artemisia* pollen is a major important outdoor allergen in China. It has been verified as an allergen by nasal challenge and bronchial provocation tests, and these allergens have been shown to occur not only in its pollen but also in its leaves and stems. Two national allergenic pollen surveys have been conducted in China. The main allergenic* Artemisia* species in mainland China were recorded and described by color photos. Immunological changes from* Artemisia* pollen-allergic subjects were studied, including Th1 and Th2 balance, basophils, HLA-DR, and ICAM-1.

*Artemisia* pollen can trigger not only allergic rhinitis but also asthma alone or both of them. Almost half of the patients with autumnal pollen allergic rhinitis developed seasonal allergic asthma within 9 years. IDT and sIgE detection are well correlated with each other in* Artemisia* pollinosis diagnoses. Specific IgE reactivity towards the major mugwort allergen Art v 1 is a good indicator for Art v sensitization. In 1989, asymptomatic* Artemisia* pollen asthma patients were selected for a double-blinded oral immunotherapy trial. Recently, a Stage I clinical SLIT trial for* Artemisia annua *L. was permitted by the Chinese Food and Drug Administration for one Chinese pharmaceutical company.* Artemisia* immunotherapy could induce the desensitization of basophils, and basophil desensitization may play an important role in immunotherapy mechanisms.

## Figures and Tables

**Table 1:** * Artemisia* pollen season distribution in Chinese cities (July–October), unit: grain.

Province	City	July	August	September	October
Beijing	Beijing^*^	85	1773	1492	186
Tianjin	Tianjin^*^	107	632	631	42
Hebei	Baoding^*^	81	778	1852	185
Hebei	Shijiazhuang^*^	42	282	590	164
Shanxi	Taiyuan^*^	52	3203	1518	26
Shanxi	Yuncheng^*^	0	81	581	972
Inner Mongolia	Hohhot^*^	1071	1639	1088	152
Inner Mongolia	kerqinzuoyihou^*^	467	1156	896	25
Liaoning	Shenyang^*^	72	1330	327	39
Liaoning	Dalian^*^	72	554	802	169
Jilin	Changchun^*^	822	1620	210	5
Jilin	Tonghua^*^	6	385	171	6
Heilongjiang	Harbin^*^	14	1977	139	99
Heilongjiang	Jiamusi^*^	2	2178	47	24
Heilongjiang	Qiqihar^*^	18	1748	43	22
Shandong	Ji'nan^*^	31	421	1936	131
Shandong	Qingdao^*^	96	106	319	86
The Ningxia	Yinchuan^*^	377	2805	533	101
Shaanxi	Xi'an^*^	8	256	1132	431
Gansu	Jiuquan^*^	5	24	8	5
Qinghai	Xining^*^	49	1048	1673	84
Xinjiang	Urumqi^*^	10	711	1555	196
Xinjiang	Hami^*^	21	34	68	75
Tibet	Lhasa^*^	1325	1817	549	93
Henan	Zhengzhou^*^	28	35	836	249
Hubei	Wuhan^#^	23	37	221	61
Hubei	Xiangyang^#^	3	1150	1194	354
Anhui	Hefei^*^	3	62	24	8
Sichuan	Chengdu^*^	16	114	28	3
Shanghai	Shanghai^#^	8	7	207	64
Jiangsu	Nanjing^#^	2	71	643	35
Jiangsu	Suzhou^#^	0	0	58	13
Zhejiang	Hangzhou^#^	0	0	0	6
Jiangxi	Nanchang^#^	2	52	1050	45
Fujian	Fuzhou^#^	0	53	77	48
Guizhou	Guiyang^#^	0	1	1	0
Yunnan	Kunming^#^	23	13	97	324
Hunan	Changsha^#^	0	0	56	27
Guangdong	Guangzhou^#^	0	0	14	20
Guangxi	Nanning^#^	0	3	226	156

^*^Cities in north of the Yangtze River; ^#^Cities in south of the Yangtze River.

## Abbreviations

AHR: Airway hyperresponsiveness
ECP: Eosinophil cationic protein
HBDT: Human basophil degranulation test
LTP: Lipid transfer protein
ICAM-1: Intercellular adhesion molecule-1
IDT: Intradermal skin test
MP: Mugwort pollen
PUMCH: Peking Union Medical College Hospital
SDS-PAGE: SDS-polyacrylamide gel
SLIT: Sublingual immunotherapy
FEV1: The forced expiratory volume in first second.
